# Effect of Nasal Continuous Airway Pressure With and Without Surfactant Administration for the Treatment of Respiratory Distress Syndrome in Preterm Neonates

**DOI:** 10.7759/cureus.46974

**Published:** 2023-10-13

**Authors:** Husam Malibary, Hisham Nasief, Shadi Tamur, Muhammad Ashfaq, Maria Iftikhar, Ayesha Naqoosh, Khalid Khadawardi, Ammar A Bahauddin, Ahmad Alzahrani, Amber Hassan

**Affiliations:** 1 Internal Medicine, King Abdulaziz University, Jeddah, SAU; 2 Obstetrics and Gynecology, King Abdulaziz University Hospital, Jeddah, SAU; 3 Department of Pediatrics, College of Medicine, Taif University, Taif, SAU; 4 Pediatrics, National Institute of Child Health, Karachi, PAK; 5 Pediatric Medicine, Sir Ganga Ram Hospital, Lahore, PAK; 6 Social and Preventive Pediatrics, Sir Ganga Ram Hospital, Lahore, PAK; 7 Obstetrics and Gynecology, Umm Al-Qura University, Makkah, SAU; 8 Department of Pharmacology and Toxicology, College of Pharmacy, Taibah University, Madinah, SAU; 9 European School of Molecular Medicine, University of Milan, Milan, ITA; 10 Translational Neuroscience Lab, CEINGE-Biotecnologie Avanzate, Naples, ITA

**Keywords:** preterm neonates, surfactant administration, pulmonary surfactant, nasal continuous positive airway pressure, respiratory distress syndrome

## Abstract

Background

Neonatal respiratory distress syndrome is a common cause of respiratory distress in newborns, often resulting from a lack of surfactant production or premature lung breakdown. The objective of this study was to compare the effect of nasal continuous airway pressure with and without surfactant administration for the treatment of respiratory distress syndrome in preterm neonates.

Methodology

A comparative analytical study was conducted on 100 neonates (group A continuous positive airway pressure (CPAP) with surfactant = 50 vs. group B CPAP only= 50 ). The group was allocated to the patient according to sequence. In group A, the neonates were given surfactant by the INSURE (intubation, surfactant, extubation) technique via an endotracheal tube with a single dose of 100 mg/kg/dose within the first hours of life followed by CPAP. In group B, the neonates were given only CPAP after birth. At follow-up after 24 hours, pH, pCO_2_, pO_2_, positive end-expiratory pressure (PEEP), and FiO_2_ were documented. All information was recorded on a predesigned questionnaire and results were subjected to statistical analysis to determine the significance of observed differences. Collected data were entered and analyzed using SPSS version 22 (IBM Corp., Armonk, NY, USA). Both groups were compared for mean pH, pCO_2_, pO_2_, PEEP, and FiO_2_ using an independent-sample t-test and effectiveness using a chi-square test. A significant difference was considered when the p-value was ≤0.05.

Results

Group A had a mean age of 4.84 ± 0.95 hours, while group B had a mean age of 5.5 ± 1.26 hours (p = 0.04). Gender distribution was similar in both groups, with 46.0% males and 54.0% females in group A, and 48.0% males and 52.0% females in group B (p = 0.841). Regarding post-treatment blood gas analysis, group A had a mean pH of 7.30 ± 0.05, and group B had a mean pH of 7.302 ± 0.07. While there was no significant difference in pO_2_ levels (p = 0.38), there was a substantial difference in pCO_2_ levels, with group A at 38.26 ± 4.35 and group B at 35.45 ± 4.36 (p = 0.02).CPAP parameters also showed a statistically significant difference in PEEP pCO_2_, with group A at 4.5 ± 0.73 and group B at 4.16 ± 0.37 (p = 0.004). After treatment, group A exhibited significant improvements in blood gas analysis and CPAP parameters compared to group B.

Conclusions

The study revealed that both CPAP with and without surfactant treatment effectively treat respiratory distress syndrome in preterm infants, with both being safe, effective, secure, and reducing side effects. However, CPAP treatment without surfactant is a non-invasive and cost-effective option.

## Introduction

Providing adequate respiratory support, particularly for premature babies, is crucial for their survival. The commonly used approach is continuous positive airway pressure (CPAP), which facilitates lung expansion and easier breathing. Neonatal respiratory distress syndrome (NRDS) is a common cause of respiratory distress in newborns, primarily affecting premature babies immediately after delivery [1]. Overall, 15% of term infants and 29% of late preterm infants develop respiratory morbidity, with higher rates in infants born before 34 weeks, with gestational age having an inverse relationship [2]. Preterm birth can result from premature membrane rupture, labor induction, spontaneous labor, or surgical delivery. To reduce respiratory distress, cautious management of pregnancies with membrane rupture should be attempted, with the likelihood increasing with gestational age [3]. One of the most frequent causes of death in the first month of birth in the United States is respiratory distress syndrome (RDS) [4].

Non-invasive ventilation and CPAP are superior to intubation and mechanical ventilation in preventing chronic lung disease in preterm-born infants. Various strategies and devices have been developed to improve this non-invasive approach and avoid mechanical ventilation. Some devices provide initial support and can provide positive pressure ventilation (PPV) if needed, while others are designed for long-term respiratory support in the neonatal intensive care unit (NICU) [5]. The ultimate goal is to provide stable, continuous airway pressure to minimize breathing work and improve gas exchange. Preterm delivery causes over 30% of global newborn mortality, with NRDS being the main cause. NRDS decreased from 20.5 per 100,000 live births to 13.4 between 2003 and 2013. Pakistan ranks fourth in Asia in preterm births, with maternal issues accounting for 15-25% of cases, with 748,100 cases annually, compared to 5-18% across 184 countries [6].

NRDS is a disorder caused by insufficient surfactant production or inactivation in developing lungs, primarily in premature neonates. This deficiency causes alveoli to collapse during expiration, leading to inadequate oxygenation and increased breathing work. Surfactant therapy has reduced mortality rates by 50% [7,8].

Infants with RDS requiring assisted ventilation should receive intratracheal surfactant within two hours of birth. Nasal bubble CPAP has gained popularity as an alternative to intubation in underdeveloped countries, as surfactant therapy is expensive and not available in public settings [9]. CPAP is the first-line therapy for RDS in newborns, providing positive end-expiratory pressure (PEEP) to improve oxygenation and reduce breathing work. It is currently the first level of intervention for preterm infants with low birth weight [10,11]. CPAP is a system consisting of a neonatal nasal cannula, a humidified oxygen source, and a calibrated plastic bottle, connected with medical tape for watertight seal and bubble detection during exhalation [12]. Surfactant is often followed by CPAP for additional therapy in neonates, with bubble nasal CPAP being successful in 76% of infants under 1,250 g and 50% of those under 750 g of birth weight [13].

Early nasal continuous positive airway pressure (NCPAP) administration after delivery reduces lung damage compared to intermittent PPV. Effective treatment for bronchopulmonary dysplasia is crucial in the extremely preterm population, especially when risk factors such as surfactant deficiency, chorioamnionitis, and increased lung water content are present. PPV at delivery determines which newborns benefit most [14].

A study found that premature infants often require CPAP for respiratory support during RDS. However, CPAP can cause nasal septum erosion, swallowing difficulties, and pneumothorax. Reducing these issues can be achieved by selecting appropriate prongs, inserting gastric tubes, and monitoring pressure. Mechanical ventilation is recommended for low-resource settings, but more extensive observations are needed to determine its long-term benefits [15]. A study on 82 neonates with RDS found that CPAP with surfactant administration significantly improved treatment response rates and hospital stays, reducing complications and enhancing respiratory function. This therapy is beneficial for patients with RDS, as it encourages recovery and reduces mechanical ventilation requirements [16].

Premature newborns often suffer from RDS, affecting their morbidity and mortality. Advanced pharmacology techniques, such as prenatal corticosteroids and postnatal surfactant therapy, have reduced RDS prevalence and severity. CPAP breathing is the primary treatment for RDS, but recent studies suggest adding pulmonary surfactant can improve outcomes. This study aims to investigate the impact of nasal continuous airway pressure, with or without surfactant administration, on the treatment of RDS in preterm neonates.

## Materials and methods

Data collection

After obtaining ethical approval (IRB-UOL-/1815-V/2022) from the Institutional Review Board Committee of the University of Lahore, Lahore Pakistan, an open-labeled randomized controlled trial study was conducted according to CONSORT guidelines on 100 neonates from the Paediatrics Department, Neonatal Intensive Care Unit, the University of Lahore Teaching Hospital, Lahore. The sample size was calculated using the effectiveness of treatment in CPAP with surfactant group (90.24%) and CPAP without surfactant group (70.73%) [16] with a level of significance of 5%, 80% power of test, and 20% expected dropout rate.

Sample selection

All preterm neonates of both genders delivered at gestational age 32-34 weeks with RDS were included in the study. Neonates with congenital abnormality at birth such as tracheoesophageal fistula, diaphragmatic hernia, choanal atresia, and neonates with birth asphyxia (APGAR score <4 at minute five) were excluded from the study.

Group assignment

Patients were randomly divided into two study groups in a ratio of 1:1. (groups A and B). A trial sequence was generated using random allocation software 2.0 and placed in sealed, numbered, opaque envelopes. A single patient’s assignment was included in each envelope. The researchers enrolled the patients, and the statistician created the random allocation sequence.

Demographic profile

After obtaining written informed consent from the parents/guardians of the parents, the patient’s history including gestational age, diagnosis, and five-minute APGAR score was noted.

Treatment allocation

Treatment assignment was done according to the group mentioned in an opaque sealed envelope according to the sequence number of patients without blinding. Baseline variables, i.e., pH, pCO_2_, and pO_2_ were noted by arterial tap and CPAP parameters were set as PEEP (4-8 cmH_2_O), FiO_2_ (40-60%) before giving surfactant and application of CPAP. In group A (CPAP with surfactant), the neonates were given surfactant using the INSURE (intubation, surfactant, extubation) technique via an endotracheal tube with a single dose of 100 mg/kg/dose within the first hours of life followed by CPAP. In group B (CPAP without Surfactant), the neonates were only administered CPAP after birth. At follow-up after 24 hours, pH, pCO_2_, pO_2_, PEEP, and FiO_2_ were documented and labeled in the form of effectiveness. Duration of hospital stay was noted. All information was recorded on a predesigned proforma and results were subjected to statistical analysis to determine the significance of observed differences.

Data analysis

SPSS version 22 (IBM Corp., Armonk, NY, USA) was used to enter and analyze the collected data. Quantitative data including gestational age, weight, hospital stay, pH, pCO_2_, pO_2_, PEEP, and FiO_2_ were presented in the form of mean ± SD. Qualitative data including gender and effectiveness were presented in the form of frequency and percentage. Both groups were compared for the mean difference of pH, pCO_2_, pO_2_, PEEP, and FiO_2_ using an independent-sample t-test and effectiveness by using a chi-square test. To account for effect modifiers, the data were stratified for gestational age and gender. To compare the effectiveness in stratified groups, the post-stratification chi-square test was used. Results were considered significant if the p-value <0.05.

Outcome and utilization

This study can provide us with data regarding the effectiveness of CPAP with and without surfactant administration. We may utilize this low-cost CPAP in the treatment of premature neonates with RDS in low-resource settings.

## Results

In this study, 100 neonates fulfilling the selection criteria were enrolled. In group A, the mean age was 4.84 ± 0.95 hours, while in group B, the mean age was 5.5 ± 1.26 hours (p = 0.04). There were 23 (46.0%) males and 27 (54.0%) females in group A and 24 (48.0%) males and 26 (52.0%) females in group B (p = 0.841). The mean gestational age in group A was 32.7 ± 0.70 while in group B was 2.9 ± 1.83 (p = 0.592). The blood gas analysis showed a significant difference in pH level (p ≤ 0.05), and pCO_2_ and pO_2_ showed insignificant differences (p ≥ 0.05). There was a statistically insignificant difference in CPAP (p > 0.05) (Table 1).

**Table 1:** Baseline characteristics of study participants. ^a^: independent-sample t-test; ^b^: chi-square test. Group A: continuous positive airway pressure with surfactant. Group B: continuous positive airway pressure without surfactant.

Variables	Group A (n = 50), f (%) or mean ± SD	Group B (n = 50), f (%) or mean ± SD	P-value
Age (hours)	4.84 ± 0.95	5.5 ± 1.26	0.04^a^
Gender			
Male	23(46.0%)	24(48.0%)	0.841^b^
Female	27(54.0%)	26(52.0%)	
Gestational age in (weeks)	32.7 ± 0.70	2.9 ± 1.83	0.592^a^
Blood gas analysis			
pH	7.19 ± 0.06	7.23 ± 0.05	0.04^a^
pCO_2_	51.64 ± 5.63	53.0 ± 4.30	0.178^a^
pO_2_	58.70 ± 6.04	58.1 ± 5.70	0.611^a^
CPAP parameters			
PEEP	7.0 ± 0.014	7.32 ± 0.68	0.001^a^
FiO_2_	67.1 ± 4.85	69.8 ± 4.28	0.004^a^

A comparison of post-treatment blood gas analysis reported that the mean pH in group A was 7.30 ± 0.05 and in group B was 7.302 ± 0.07. The mean pCO_2_ in group A was 38.26 ± 4.35 and in group B was 35.45 ± 4.36 (p = 0.02). The mean pO_2_ in group A was 73.8 ± 74.5 and in group B was 74.5 ± 4.54 (p = 0.38). A statistically significant difference was reported in pCO_2_. The CPAP parameters showed a statistically significant difference in PEEP pCO_2_ (group A = 4.5 ± 0.73 vs. group B = 4.16 ± 0.37; p = 0.004). However, FiO_2_ remained the same (p = 0.668) (Table 2).

**Table 2:** Comparison of blood gas analysis and continuous positive airway pressure parameters after treatment among the study participants. Independent-sample t-test. Group A: continuous positive airway pressure with surfactant. Group B: continuous positive airway pressure without surfactant.

Variables	Group A (n = 50), mean ± SD	Group B (n =50), mean ± SD	P-value
Blood gas analysis			
pH	7.30 ± 0.05	7.302 ± 0.07	0.88
pCO_2_	38.26 ± 4.35	35.45 ± 4.36	0.02*
pO_2_	73.8 ± 74.5	74.5 ± 4.54	0.382
CPAP parameters			
PEEP	4.5 ± 0.73	4.16 ± 0.37	0.004*
FiO_2_	39.0 ± 2.25	38.8 ± 2.38	0.668

**Figure 1:**
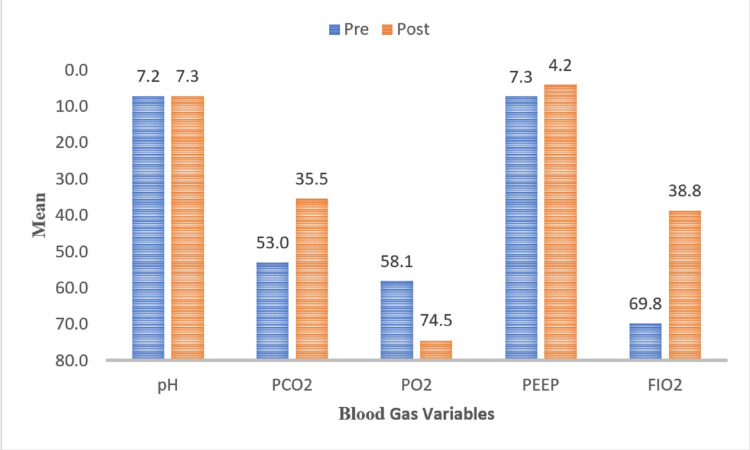
Comparison of pre- and post-blood gas analysis and continuous positive airway pressure parameters within the surfactant group.

The post-treatment blood gas analysis and CPAP parameter in CPAP with surfactant group are presented in Figure 1. The results revealed that after treatment there was a significant improvement in blood gas analysis and CPAP parameter in group A (p < 0.05).

## Discussion

RDS is the most common respiratory complication that occurs in neonates after preterm birth. It usually manifests within hours after birth, most frequently right after delivery. Insufficient surfactant production or surfactant degradation in the presence of immature lungs are the two main contributing factors to NRDS. In the management of newborn care, the discovery of surfactants has been significant as the therapy precisely addresses surfactant deficiency and alters the etiology and outcome of RDS [9]. In neonates with RDS, who require respiratory support, non-invasive ventilation is more often used compared to mechanical ventilation. Non-invasive ventilation includes several ventilation techniques. NCPAP and early surfactant therapy should be initiated at delivery in preterm infants with RDS [16]. This study compared the effectiveness of NCPAP with and without surfactant in preterm neonates with RDS.

A total of 100 neonates (50 in each group) fulfilling the selection criteria were enrolled in this study. In group A, the mean age was 4.84 ± 0.95 hours, while in group B, the mean age was 5.5 ± 1.26 hours (p = 0.04). There were 23 (46.0%) males and 27 (54.0%) females in group A and 24 (48.0%) males and 26 (52.0%) females in group B (p = 0.841). The mean gestational age in group A was 32.7 ± 0.70 while in group B was 2.9 ± 1.83 (p = 0.592). There was no statistically significant difference in blood gas analysis (p > 0.05).

The results of this study revealed that both treatment methods enhanced the effectiveness but with insignificant differences. Infants with RDS are now treated with CPAP along with a combination of supportive treatments to reduce the symptoms of hypoxia. However, due to its high technical maintenance and requirements, mechanical ventilation is associated with more complications which can affect recovery and prognosis [17].

Currently, PPV, surfactant, and supportive therapy are the major treatment modalities for RDS. A single-centered, randomized control trial conducted in a tertiary NICU from January 2008 to December 2010 at Daping Hospital, Third Military Medical University included 179 preterm infants who were diagnosed with RDS. The infants were randomly allocated into two groups (CPAP with surfactant and control). The study revealed insignificant differences in blood gas analysis in both groups (p > 0.05). There were no associated complications in both groups (p > 0.05) [18]. These findings are consistent with those of the current study, highlighting that CPAP with and without surfactant administration had insignificant differences in effectiveness.

A study by Wang 2018 [19] aimed to determine the effect of pulmonary surfactant in the treatment of RDS in infants. Patients were randomly allocated into the surfactant and control groups. The study revealed that after six hours of treatment, there was a significant difference in the hospital duration, blood gas analysis, and duration of ventilation in the surfactant and control groups. It concluded that the use of surfactant therapy improves the function of the lungs and reduces mechanical ventilation and hospital stay [19]. In this study, after treatment, the children’s pulmonary function improved markedly, ventilator parameters decreased significantly, and the degree of alveolarization increased significantly, indicating that treatment with pulmonary surfactant had a major clinical effect.

Another intervention study evaluated the effectiveness of surfactant with CPAP enrolling a total of 50 infants with RDS. The patients’ blood gas analysis, mechanical ventilation parameters, and pulmonary arterial pressure were observed. The study revealed that the PaO_2_, pH, and oxygen levels after treatment in the intervention group were higher than those in the control group. The findings revealed that surfactant therapy has a significant effect on ventilation and lung functionality along with being clinically significant [20]. The CPAP and surfactant replacement therapy appear to be the most effective methods for treating respiratory distress in preterm infants with RDS in low- and middle-income nations [21].

The literature suggests that non-invasive ventilation and CPAP are more effective than intubation and mechanical ventilation in preventing chronic lung disease in preterm-born infants. Various strategies and devices have been developed to improve this non-invasive approach. While some devices are affordable and designed for low-income countries, others are complex and expensive, offering features such as bilevel pressures and high-frequency oscillating pressures. Surfactant should be administered as early as possible in the course of RDS when it becomes clear that mechanical ventilation is likely to be required. This likelihood varies from infant unit to infant unit and depends on the comfort and experience of the nursing staff with CPAP. The reduction in morbidity associated with RDS is related to the decrease in the use of mechanical ventilation through a combination of maternal prenatal steroid treatment and early intervention via INSURE [22]. Another study included infants with different lung disease severity and gestational age. In the study, patients with respiratory distress were randomized to receive either CPAP only or CPAP with surfactant. The study enrollment period ranged from 30 minutes to 72 hours after delivery. The use of surfactant therapy in neonates using CPAP was found to reduce the risk of mechanical ventilation. The results revealed that the administration of surfactant therapy minimizes the need for mechanical ventilation from 70% to 50% [23].

Another study conducted on the use of surfactant therapy reported the requirement of mechanical ventilation in infants with RDS reduced from 68% to 25% [24]. These findings also show evidence for the use of surfactant therapy for improving RDS which was dissimilar with the current study. This study reported that both treatment of CPAP with and without surfactant is equally effective. Rojas et al. randomly assigned preterm infants who showed signs of respiratory distress in the first hour after birth and administered supplemental oxygen in the delivery room to either the treatment group, which included intubation, very early surfactant with CPAP, or the control group treated with only CPAP. There was a notable difference between the treatment group who received surfactant after the first hour of life (12% versus 26%) [9].

In preterm neonates, the management of RDS ventilated predominantly with NCPAP was recently studied in India. The study investigated the role of early surfactant administration. All neonates of 28-33 weeks of gestational age who received NCPAP within the first two hours of life were randomly assigned to either early routine surfactant administration using the INSURE or late selective surfactant administration. The main outcome was the need for mechanical ventilation within the first seven days of life. In 153 infants randomly assigned to the early or late surfactant group, the need for mechanical ventilation was significantly lower in the early surfactant group [25].

Another randomized controlled trial was conducted on the role of surfactant and non-invasive mechanical ventilation on infants with RDS. The study reported that non-invasive respiratory support was a significant treatment method in the early management of RDS. CPAP with a combination of surfactants is an effective and safe alternative for routine ventilator support and decreases the risk of mortality and associated complications such as bronchopulmonary dysplasia. The non-invasive ventilator support is also a safe and cost-effective approach for the treatment of RDS [26]. The findings of this study also support the current study which proves that both treatments are equally effective for treating RDS.

Gozde et al. aimed to investigate the role of early use of surfactant with the Take Care/INSURE method in comparison to mechanical ventilation. All preterm neonates with RDS and a gestational age of 32 weeks were enrolled in the study. The results revealed that within the first 72 hours of life, the requirement of mechanical ventilation was lower surfactant with the Take Care/INSURE method. The study proved that this method significantly reduces respiratory support and has fewer side effects [27].

More randomized, controlled, and powered trials with adequate follow-ups are needed to better determine the true efficacy and long-term adverse effects of both treatment methods. The limitation of the current study was the small sample size because the number of patients was not sufficient to clearly explain the significance of both treatment methods and made it difficult to determine cause-and-effect relationships among the variables studied.

## Conclusions

The treatment of RDS and the prevention of the development of chronic lung disease have always been crucial goals. Numerous studies conducted over the past decade have shown that non-invasive respiratory support is a safe and reliable method of preventing RDS and not all preterm infants require surfactant treatment. This study revealed that CPAP with and without surfactant treatment have equal effects in treating RDS. However, CPAP without surfactant administration is a non-invasive and cost-effective method for treating RDS in preterm neonates. It is a suitable treatment method for low socioeconomic status countries.
